# Dr. George Gregory's Elements of the Theory and Practice of Physic (2d Ed.)

**Published:** 1826-10-01

**Authors:** 


*Elements of the Theory and Practice of Physic, designed for the Use of Students.* By George Gregory, M.D. &c. &c. Second Edition, with Additions and Amendments. 8vo. pp. 663. London. Burgess & Hill, &c. 1825.

These Elements have reached a second edition, and this may be deemed a proof of their merits. The first part, or that which embraces the consideration of acute diseases, has already been noticed, and it is the second, or the discussion of chronic affections, that now engages our attention.

It was not the object of the author, like Dr. Good, to write a complete system of theoretic and practical medicine; nor, like Dr. Dawson, only to express general observations on particular diseases; but to produce an elementary work for the use of students, and in this we think he has been very successful. Dr. Gregory is master of a pretty style, and this is no mean matter in interesting readers of every description. We cannot, indeed, bestow a higher epithet than pretty, since that style can have no great pretensions either to correctness or elegance, which has "referable" for referrible, "sanguine" for sanguineous, "two first" for the first two, "spiculæ" for spicula, and "a sort of death beginning," *cum multis aliis.** Yet still the style is pretty, just as a body is called round although it presents numerous inequalities.

The second part, on chronic diseases, occupies three hundred and thirty pages of closely printed matter, and, after three pages of preliminary remarks, it comprises five classes, an appendix, and formulæ medicamentorum. We will state the distinctive appellation of every class; each of which is divided into a certain number of chapters.—Class I. Chronic diseases of the encephalon.—Class II. Chronic diseases of the thorax.—Class III. Chronic diseases of the chylopoietic viscera.—Class IV. Chronic diseases of the urinary and uterine systems.—Class V. Chronic constitutional diseases.

We shall now offer to the reader a specimen of this elementary work, not that it is one of the best, but because it may be productive of utility in a practical point of some importance.

"Hooping cough is not described by any of the Greek, Roman, or Arabian authors. It is impossible to suppose that a disease so strongly marked as this, could have escaped the attention of the ancient physicians, had it then existed. We must presume, therefore, that it was not known in Europe before the thirteenth, or perhaps even the four-

---

* Among many others "*lesser*" for less; and, as this is a very common error it may be useful to expose it. He who writes "*lesser*" for less imagines that it is the comparative of the adjective *less*, whereas it is the comparative of the adjective *little*:—thus *little, less, least;* and not *less, lesser, least.*—"Lesser" says Johnson, "is a barbarous corruption of *less*, formed by the vulgar from the habit of terminating comparisons in *er*."—Even Addison falls into this error,

"Attend to what a *lesser* muse indites."

*Worser* is not more incorrect than *lesser*; but sounds more barbarously because not so frequently employed.

"Changed to a *worser* shape thou cans't not be."
Shakspeare, 1 *Hen.* VI.

For farther information see the grammar of Bishop Lowth.—*Rev.*

teenth century. It was first accurately described by Dr. Willis* in 1664. The most complete treatise on the disease which has since appeared is that of Dr. Watt of Glasgow,† in which the student will find a copious account of the opinions of the best authors.

"The phenomena which hooping cough presents, as well in its origin as in its subsequent progress, may be thus briefly described. It begins with the common symptoms of catarrh, from which indeed it cannot be distinguished by any known criterion for the first week. It has been observed, that the usual catarrhal symptoms are here accompanied with a more than ordinary disposition to sleep, and those which denote general fever are seldom very strongly marked. About the end of the second, or beginning of the third week, the symptoms undergo a remarkable change: the fever declines, and appetite returns; but the cough continues, and occurs in paroxysms of extraordinary violence. The child struggles for breath, and appears in danger of suffocation, until relieved by the long and full inspiration known under the name of the *back draught*, or hoop. The fit of coughing continues for several minutes, and is commonly terminated by expectoration of mucus, sometimes by vomiting, and occasionally by bleeding at the nose, or an epileptic paroxysm. In very bad cases, even this relief is denied to the little patient, whose efforts end only with his complete exhaustion. It is distressing to witness the attempts made to expectorate. The child appears conscious of the benefit which is thus afforded to him, and he continues coughing until expectoration is effected.

"The fits vary much in frequency. In mild cases they do not occur more than three or four times a day. In severe ones, they harass the patient every half hour. It is very rare to find them recurring at regular intervals. They are often brought on by exertions of body, or emotions of mind. It is common, therefore, to find the child averse from moving or speaking. He is often aware of the approach of the fit, and lays hold of any thing near him for support. He finds relief by stooping forward, and by support given to the head and back.

"When once the disease has assumed its regular form, the appetite is good, and this is strikingly displayed in the craving for food, which comes on when the fit terminates by vomiting. The tongue is always *clean and moist*. There is no difficulty of breathing in the intervals of the fit. Permanent dyspnœa betokens something more than mere hooping cough,—probably an inflammatory condition of the bronchial membrane. The bowels are seldom affected. It is very common to find children with hooping cough complaining of a *tensive* pain of the forehead, and in severe cases this is obviously an *urgent* symptom, and one which demands attention in reference to practice.

"The further progress and duration of hooping cough are subject to great variety. In its mildest form it generally lasts two or three months; and when severe, is often protracted to six or seven. Even after it has

"* Pathologia Cerebri et Nervosi Generis, cap. 12."

"† Treatise on the Nature, History, and Treatment of Chincough, 1813."

wholly ceased, or nearly so, an accidental exposure to cold has occasioned its return. Under the most favourable circumstances the decline of the disease is very gradual, and almost imperceptible. It happens, however, but too frequently, that the latter stages of the disease are attended with a formidable train of evils. In some cases a convulsion fit occurs in one of the paroxysms, and carries off the patient when the practitioner is least prepared for it. In other cases, from exposure to cold, pneumonic symptoms supervene, and the child either dies with his lungs gorged with blood, or the foundation is laid for a species of infantile phthisis.* In a third set of cases, hooping cough brings on genuine hydrocephalus, and the child dies in a state of coma. This might oftener be anticipated, when we reflect with what force the blood is driven upon the brain, and how much its return is retarded, during a severe fit of coughing. But of all the modes by which hooping cough proves fatal, the most common is that by *marasmus and infantile fever.* The child, after a continuance of the disease for a certain time, from causes not well understood, loses his appetite, emaciates rapidly, becomes hectic, and dies, *apparently* from pure exhaustion.†

"The danger is not proportioned to the age of the patient. A child of two or three *months* old will struggle through the complaint as well as another of two or three years. When it attacks weakly or scrofulous children, or those labouring under some other disease, it is apt to prove severe, tedious, and therefore dangerous. When hooping cough begins late in the spring, it is commonly milder than when its approach is towards the beginning of winter. It is always most destructive in cold climates, and in cold and damp seasons.

"The appearances on dissection correspond with the views which have been given of the modes by which this disease proves fatal. Dr. Watt has described several cases in which there were found the clearest proofs of acute bronchial inflammation, conjoined with more or less *congestion* in the substance of the lungs. In some which have been recorded, serous effusion within the ventricles of the brain has been the predominant morbid appearance; while to myself and to many others it has occurred to witness numerous instances, in which, on examination, nothing preternatural has been observed in either of the three great cavities of the body.

"Hooping cough, though sometimes met with in adults, is for the most part the disease of early life. It is often epidemic. Few children escape it; but it rarely, if ever, is known to occur more than once in the course of life. From these and other facts which might be adduced,

---

"* The deaths by hooping cough recorded in the London bills of mortality are always very numerous, averaging not less than five hundred annually. In 1822, they amounted to seven hundred and fifty-seven, exceeding the deaths by small-pox."

"† The pathology of this, and of the other varieties of *infantile hectic*, is very little known. An attempt will be made to investigate the subject in a subsequent chapter."

a reasonable presumption exists, that it has its origin in a *specific contagion*, which, like those of the influenza and measles, has a direct determination to the membrane of the bronchia, though it is not, like them, essentially linked to fever. The contagion of hooping cough appears to be communicated with great facility. When once it gets entrance into a family, it generally attacks every child.

"Different opinions have been entertained regarding the precise nature of hooping cough. It was originally considered as a spasmodic disease, allied in its more obvious features to asthma and chorea, but acknowledging also many of the laws of convulsive diseases generally. This simple and very satisfactory explanation of the pathology of hooping cough has latterly been called in question; and it has been confidently maintained that it is an affection of an inflammatory kind, closely allied to the ordinary varieties of bronchitis. In favour of this opinion it has been argued; 1. that common winter cough frequently shows a strong disposition to spasmodic exacerbation; 2. that all the more important *sequelæ* of hooping cough are of a decidedly inflammatory character; and, 3. that inflammatory affections of another mucous membrane (catarrh, and cynanche maligna) are induced by the operation of a specific contagion. To these arguments it may be replied, that they point out a strong *tendency* in this disease to inflammation, which the practitioner will do well to keep constantly in view; but an impartial observer will not fail to appreciate those more numerous considerations which associate it with the class of spasmodic diseases." 441.

Now we are by no means converts to the opinions here promulgated; and, with due caution and reserve, are more inclined to coincide in the sentiments of Dr. Dawson, who, in a recent practical work evidently resulting from considerable experience, has treated this subject very elaborately. It is not our intention, and it would be against our principles, to institute any comparison between Dr. Dawson and Dr. Gregory, or to praise either of these physicians at the expense of the other:—no; we have a higher object in view, which is to call the attention of practitioners in general to the right consideration, and proper treatment of an always distressing, and too frequently dangerous, disease. The discussion we are about to enter into will, we fervently hope, stimulate observing and experienced men to communicate their opinions, unquestionably valuable, to the pages of some of the respectable periodical journals, with the object of ascertaining whether Dr. Dawson or Dr. Gregory be the safest guide, or the most faithful observer. To us, abstractedly speaking, it imports very little;—to the community almost every thing. He that has had a beloved child, the only remains, perhaps, of a much loved object, suffering under severe hooping-cough; who has seen that child in all its strength, and all its weakness; who has beheld it coughing with agony, gasping for breath, clinging to chairs and tables for support; and who has witnessed his bloodshot eyes, and the blood gushing from his nostrils, or streaming from his mouth, will pardon such a discussion, if even he take no interest in it.—That we may do full justice to Dr. Dawson, and give additional publicity to that physician's doctrines, as well as place them on record for immediate perusal, and subsequent reference, we will subjoin them in a somewhat abridged form, at the bottom of this page, as a note.*—Now it is incontrovertible that in assuming a peculiar kind of inflammation of the glottis to constitute the primary stage of pertussis, Dr. Dawson could have no ends to answer except those of truth, since he candidly acknowledges that twenty years ago Sir Astley Cooper called his attention to that simple fact. Let it not be forgotten that the opinion of this gentleman on such a point is exceedingly influential. If Sir Astley had been a prac-

---

* Hooping cough consists, not in spasm, but in an inflammation of the glottis, which either remains there, or involves the larynx, trachea, and lungs. The specific nature of this inflammation, like other specific ones, satisfactorily explains why pertussis is often contagious, and why, in its effects as well as in its duration, it widely differs from common inflammatory affections."—"The inflammation may be subacute or acute, passive or chronic; and according to its kind and degree, so will the cough, depending on such inflammation, be mild or severe, short or long continued, cure itself spontaneously, or terminate unhappily. If the inflammation be subacute, it will proceed favourably to health; if passive or chronic, it may last a considerable time without danger; and if highly acute, the inflammation may, and often does, reach the lungs, and destroy life like pneumonitis, for such then is the disease, with pertussis superadded. The duration and event of hooping cough correspond with what is here advanced:—Sometimes it extinguishes life in a few days; and occasionally it obtains for several weeks; and not unfrequently it is protracted to the seventh or eighth month."—"The fever which attends hooping cough varies in degree, being sometimes slight, and disappearing in a week; and, occasionally, severe and long continued, with oppression, sickness, and want of appetite. When the cough is formed, the paroxysm consists of a number of short expirations in rapid succession, so as to induce a sense of suffocation, which is relieved, for an instant, by a violent, full, and *hooping* inspiration;—then the expirations recommence; and thus, in alternate succession, does the paroxysm, always most frequent and distressing during the night, continue until it terminate by a discharge of phlegm, the contents of the stomach, or hæmorrhages from the nose or lungs."—"If the inflammation be confined to the glottis, the health does not commonly suffer: the appearance is seldom altered: there is little fever, and in a few weeks the inflammation slowly subsides, the characteristic *hoop* departs, and recovery follows as if from catarrh. In other instances the inflammation, still confined to the glottis, and slightly implicating the larynx, becomes decidedly chronic; and the paroxysms, with discharges of phlegm, food, and blood, attended by a swollen countenance, continue for many months. Even here no great danger is to be apprehended, because the inflammation evinces no dispositising physician, if he had been, like Dr. Gregory or Dr. Dawson, a systematic writer on physic, he might have entertained a particular theory on hooping cough, or any other disease, and have been most anxious to demonstrate its correctness; but, being an eminent surgeon only, it is evident that he could take no interest in an affection foreign to his pursuits, and could alone have formed, and promulgated the opinion just mentioned from numerous dissections performed by his own hands. But Sir Astley Cooper simply stated the fact itself; and the assertion that it is a peculiar kind of inflammation, and divisible into subacute or acute, passive or chronic, are the results of Dr. Dawson's experience and reflection for a period of twenty years. That a peculiar inflammation does frequently invade the air-passages is a self evident proposition; and Dr. Dawson forcibly urges that while the inflammation of croup produces a dangerous exudation of coagulable lymph, that of chronic tracheitis only causes a secretion of purulent fluid. Every sensible

---

tion to spread;—there is no serious affection of the general health—no fever—no convulsions—no anasarcous swellings—the child manfully grapples with the disease, and will not be overpowered. But more unhappy cases meet our view, and most generally in extreme youth and debilitated frames. Here every symptom is unfavourable. The child is exceedingly feverish, even in the intermissions. The paroxysms are frequent, long, and violent; there is considerable exhaustion of the system; the sufferer pants for breath; even after the paroxysm it does not breathe freely, and its face is flushed or pale alternately: there is no appetite—no activity—but thirst, and unwillingness for motion; and all this may occur within the first twenty days. Where the inflammation of the glottis is most acute: disdaining its own narrow limits, it seeks to extend them by rapidly travelling over the larynx, trachea, and lungs. Now the patient, in addition to pertussis, has pneumonitis to contend against, of which it presents every symptom. If the disease proceed unchecked, the breathing becomes still more difficult, the paroxysms abate, convulsions sometimes occur, and death ensues from copious effusion into the lungs."—*Dawson's Nosological Practice of Physic*, p. 125, *et sequent. London*, 8vo. 1824.

The above Note is but, at best, an abridged statement of the opinions of Dr. Dawson; and in this respect we have given Dr. Gregory a decided advantage over him, since his narrative is altogether unbroken. Exceedingly anxious to reflect light on this important subject we, this evening, asked the candid opinion of a very eminent general practitioner, with whom we were in attendance, and who probably, during the last eighteen years, has seen as much of hooping cough as any man now living. He answered us thus,—"I think it is always inflammatory, but certainly blood-letting does not control the disease as it commonly does other inflammations; and I suspect this is owing to some peculiarity in the inflammation itself."—It is worthy of notice that this observation, and the doctrines of Dr. Dawson receive great confirmation of their truth from an opinion, recently promulgated by Dr. Armstrong, that liberal venesection is injurious in acute bronchitis. Still, however, when inflammation begins to spread from the glottis blood-letting is of marked benefit in pertussis.—*Rev.*

and observing writer has admitted that hooping cough is very often of an inflammatory nature; but the point at issue is whether it is always inflammatory; and whether this inflammation, in the first instance, invariably assails the glottis; since these are the leading principles, which Dr. Dawson has so strenuously laboured to circulate, and to establish. Innumerable dissections have revealed the consequences of inflammatory action in the lungs, and their appendages, of the victims to pertussis; and, under such complicated mischief, it is easy to conceive, that the state of the glottis might have eluded the notice of common observers, although it could not, and did not, escape the scrutinizing eyes of the first surgeon of this, or perhaps of any other age. Even Dr. Gregory acknowledges that hooping cough begins exactly like a catarrh; and surely if this be not inflammation, it is, at least, very much in the spirit of it. Again, he says, according to the London bills of mortality, five hundred die annually of this disease; and that, in 1822, they amounted to seven hundred and fifty-seven! Need we go farther after such appalling evidence?—What else but a singularly insidious, and little suspected inflammation, of a dangerous nature, because implicating an important out-let, could occasion so unprecedented, so frightful a loss of human life?—No common causes could be adequate to the production of such wide-spreading mortality in a single metropolis!—When Dr. Gregory gravely observes that numerous considerations associate hooping cough with the class of spasmodic diseases, we fear he departs from his general accuracy, and good judgment. That spasm does attend this affection few will deny; yet who does not see that such spasm is the mere consequence of irritation, just as spasm arises from an irritable urethra, or from the introduction of a bougie.—Here our task ends; with every possible caution and deserved courtesy; with every abstinence, either from praise or censure, we have attempted to place the opinions of Dr. Gregory and Dr. Dawson on hooping cough, as it were, in juxta-position; and, without having committed ourselves farther than truth demanded, we now cheerfully leave this important subject to the consideration of the profession at large, which will, doubtless, sooner or later, decide most justly.

One more interesting extract, and then we will hasten to a conclusion.

"1. In the most usual form of apoplectic seizure, the patient falls down *suddenly*, deprived of sense and motion, and lies like a person in a deep sleep. He neither hears, nor sees, nor feels. Unconscious of every thing around him, he is alike insensible to the exertions of his medical attendants, and the anxieties of his friends. The suddenness of the attack is that feature of the disorder which most immediately impresses itself upon the notice of observers; and being so very general, the disease has from this circumstance in all ages received its name.

"2. The second form of apoplectic seizure commences by a sudden attack of violent pain of the head, accompanied with paleness of the face, sickness at stomach, vomiting, and transient loss of recollection. The patient, in some instances, falls down in a state resembling syncope, but recovers in a few minutes, and is able to walk. After a few hours, however, the head-ache continuing, he becomes oppressed, and *gradually* sinks into perfect coma.

"3. The third form of apoplectic seizure begins with a sudden attack of *palsy* of one side, with loss of speech, which, after the lapse of some hours, passes gradually into apoplexy.

"In whichever way the apoplectic fit commences, there are certain appearances presented during its continuance, which merit attention. The pulse, at first, is commonly small and irregular; but as the system recovers from the shock, the pulse becomes full and strong, and is generally slower than natural. Respiration is much embarrassed, being always slow, and occasionally irregular. In all the severer degrees of the disease, this laborious breathing is accompanied by stertor; and a frequent appearance is that of foam, or frothy saliva, excreted from the mouth, and blown away from the lips with considerable force. This latter symptom has always been looked upon as indicative of the greatest danger.

"The skin is commonly warm, and bathed in a copious perspiration. In the worst cases of the disease, a cold clammy sweat has been observed. The face is generally pale; the cornea dull and glassy; and the pupils permanently dilated. The teeth are closely clenched; and the power of swallowing, though seldom wholly lost, is for the most part so much impeded, as to oppose the most serious obstacles to the administration of remedies. The bowels are torpid, as is usual in all cases of cerebral oppression, and they resist the action even of powerful cathartics. If blood is drawn from the arm, the coagulum is commonly firm; and Sir Gilbert Blane has noticed, that it is in most instances covered with the inflammatory crust.

"The duration of the apoplectic fit varies from two or three hours to as many days. Thirty hours may be called the average duration of those cases which have fallen under my own observation. Instances, indeed, are on record of *sudden death* from apoplexy; but in many of these there is reason to suspect, that the immediate cause of death was rather to be found in some affection of the heart, or large vessels in its neighbourhood, than in injury to the brain. Genuine apoplexy, commencing in the manner I have described, and attended with all the symptoms just enumerated, almost always ends fatally. When a recovery, either perfect, temporary, or partial, takes place, it will usually be found that some of the more decided evidences of perfect coma have been wanting: the patient has given evidence of feeling when his limb is grasped, or the lancet used; the pupil has obeyed in a certain degree the stimulus of light; the mouth has not been firmly closed, or the power of swallowing wholly lost; there has been no stertor, or foaming at the mouth; nor were the premonitory symptoms strongly marked. Under such circumstances our prognosis may be somewhat more favourable; though it should even then be guarded by the reflection, that if recovery does take place, we must seldom expect it to be perfect. An incurable palsy may remain; or the memory may wholly or partially fail; or an imbecility of mind, approaching to mania, may be left. But besides this, in all cases where a decided apoplectic fit has been experienced, a relapse is to be dreaded, and recovery from a second attack is seldom if ever witnessed.

"The opportunities which the fatality of this disease has offered to the physician, for prosecuting his researches into its nature and seat, have not been lost; and we have accordingly a most extended record of the appearances found on dissection in apoplectic cases. Their variety is very great, and must be fully appreciated before any attempt can be made to explain the pathology of the disease. Extravasation of blood in some part of the cephalon, is by far the most common appearance, and is that which is generally to be anticipated. Such extravasation may take place between the membranes of the brain, on its surface, about its basis, within its ventricles, or in the midst of its substance. The quantity of fluid effused is as various as its situation; and the violence of the symptoms is found to bear a reference partly to the *quantity*, and partly to the particular *seat* of extravasation. An extensive effusion of blood is equally to be dreaded wherever it takes place; but a slight effusion is generally stated, and probably with justice, to be more dangerous in certain situations than in others. It is believed, for instance, to be much more alarming, and attended with more formidable symptoms, when occurring on the medulla oblongata, than in the anterior lobes of the brain.

"The next most usual appearance in those who die of apoplexy, is the effusion of serum, either upon the surface of the brain, or within the ventricles. In some cases we meet with turgescence of the smaller vessels, or of the great sinuses of the brain, but without effusion either of blood or serum.

"These are the common appearances presented on examination of those who die of apoplexy; and, considering their frequency, it is undoubtedly a surprising circumstance, that every now and then, after the most unequivocal symptoms, the head presents, on dissection, nothing morbid or uncommon." 337.

Upon the whole we are much pleased with this work, as well with the first as the second part; and have great pleasure in warmly recommending it to students, who, if they neglect to purchase it will not injure any one so much as themselves. Most honestly can we declare that we deeply regret we had no such publication to lighten our youthful labours, and to direct them into the right channel. In our opinion the author is too fond of grouping diseases, and remarkably negligent, whether designedly or not he best can tell, in noticing respectable contemporary writers, from whose pages he might have enriched his own with varied and valuable matter. Such culpable negligence, if indeed that be its proper designation, would have been scarcely excusable in a first edition; but in a second, in which, according to his own statement, there have been additions and amendments, it is strange, and passing strange. Still, however, these are but points of minor importance; and do not, in any material degree, derogate from the general merits of a truly respectable, and well executed elementary work. It is really refreshing to read the pages of Dr. Gregory; for while we read them we feel that we hold converse with a gentleman, and a man of honour;—a man who loves truth, and would scorn to gild a lie, or varnish a deception. He writes with caution, modesty, and good sense; states every thing fairly, comments on it discreetly, and draws conclusions which are commonly just. He is no ardent enthusiast, nor empty theorist;—he does not make minute divisions, nor endless subdivisions, which have no real existence;—he has no extravagant attachment to particular medicines, nor to precise modes of treatment;—he does not pretend to cure, or to have cured, diseases which have been considered incurable by the first physicians:—He describes that which he has seen, or believes to be true from indisputable testimony; and he wisely leaves to others the suspicious practice of explaining, and expatiating on, diseases not as they really are, but as they may seem to a warm and prolific imagination:—his opinions, his descriptions, his observations, and his deductions may be aptly compared with the useful labours of a faithful biographer, and not to the imposing efforts of a novel, or romance writer. Let it not be supposed that we assent to every opinion of Dr. Gregory, bow to his every theory, or submit to his every conclusion:—very far from it; but we well know that no man can produce a perfect work; since there are times when talent fails, when vigilance slumbers, and when industry flags. Such is the common lot of intellect, however acute, however cultivated; and, therefore, we judge, as we ought to judge, with a liberal mind, and unhesitatingly pronounce it an elementary work comparatively excellent, although it does, beyond all dispute, present occasional, and obvious deficiencies, and imperfections. To conceive is easy, to arrange is equally so;—to plan, in the mind's eye, a complete and faultless whole is apparently a simple task:—but where is the author who has not found it very different when he came to the trial:—when he felt the pressure of unexpected difficulties; when he experienced that time was sometimes wanting; that anxiety often precluded thought; that interruption commonly impeded execution; that to seek for books was not always to find them; that ill health would too frequently mar the best of efforts; and that a fit of mental indifference, or a paroxysm of agonizing pain would most certainly spoil a page, or render abortive an essay, which, under happier circumstances, might have created nearly universal satisfaction. In this spirit, not, we humbly hope, an ungenerous one, have we reviewed the labours of Dr. Gregory.

